# Extravasation of radiopharmaceuticals: Why report?

**DOI:** 10.3389/fnume.2023.1148177

**Published:** 2023-03-06

**Authors:** Thomas L. Morgan

**Affiliations:** Versant Medical Physics and Radiation Safety, Kalamazoo, MI, United States

**Keywords:** extravasation, radiopharmaceutical, injury, significant event, research needs

## Abstract

In this essay, I wish to discuss extravasation in the context of medical imaging and therapy with radiopharmaceuticals. Central to this discussion are two facts. First, they are easily identified, but the frequency of significant extravasations is unclear because there is no generally accepted definition of such an event. And second, there appears to be few reports of injuries from these events. The central thesis of this essay is that these events should be reported and followed so that agreement can be reached on the definition of a “significant” event which should be classified as a medical event in accordance with US Nuclear Regulatory Commission (NRC) regulations. I will also outline steps that can be taken to reduce the risk of extravasations.

## Introduction

In this essay, I wish to discuss extravasation in the context of medical imaging and therapy with radiopharmaceuticals. Central to this discussion are two facts. First, they are easily identified, but the frequency of significant extravasations is unclear because there is no generally accepted definition of a such an event. How much radioactivity must be lost to extravasation for there to be a substantial risk of serious tissue damage? And second, there appears to be few reports of injuries from these events.

The central thesis of this essay is that these events should be reported and followed so that agreement can be reached on the definition of a “significant” event which should be classified as a medical event in accordance with US Nuclear Regulatory Commission (NRC) regulations. I will also outline steps that can be taken immediately to reduce the risk of extravasations.

## Frequency of extravasations and sequalae

Extravasation is the inadvertent leakage of fluids or medications out of a vein or artery into surrounding tissues. As discussed by the NRC in a recent report of a subcommittee of the Advisory Committee on the Medical Use of Isotopes (ACMUI), an extravasation does not meet the intent of the medical event reporting requirement because it is not an “error” that represents a breakdown of the licensee's program for ensuring that byproduct material or radiation from byproduct material was administered as required by an Authorized User—it is not considered as the wrong route of administration (1). Consequently, such an event is not currently on the list of reportable medical events found in NRC regulations (10 CFR 35.3045).

European basic radiation safety standards (2) do not address extravasations directly. The responsibility for defining a significant accidental or unintentional exposure event rests with the competent authority of the country where the event occurred.

Personnel who handle radioactive materials in hospitals and nuclear medicine imaging facilities typically receive radiation safety training that includes definitions of radiological medical events and the responsibility to report such events to appropriate regulatory agencies. There are no requirements in regulations or standard clinical practice to follow patients after diagnostic nuclear medicine scans. Since extravasation is not a reportable event, it is not typically discussed in the radiation safety training. Personnel are not held accountable to report such an event to the radiation safety officer. Presumably, it is left to the physician who prescribed administration of the radiopharmaceutical to manage the consequences in accordance with existing policies and procedures, if any.

I have been the designated radiation safety officer (RSO) at a large community hospital in California and two university medical centers and hospitals in New York. During my 20 years of tenure at these institutions, tens of thousands of patients have undergone diagnostic medical imaging studies and therapeutic administrations of radiopharmaceuticals. During this time, no extravasations were reported to me, and I was not made aware of any reports of tissue reactions at injection sites. In addition to membership on radiation safety committees I, as the RSO, was a member of the radiology departments' quality assurance committee at two of these institutions. Extravasations were not reported to or tracked by these committees.

It is a well-established fact that extravasations of drugs and fluids occur during the normal course of medical care. There are reports in the literature of events involving radiopharmaceuticals. A recent review by van der Pol et al. summarized published reports of radiopharmaceutical extravasations (3). Thirty-seven publications reported 3,016 cases involving diagnostic agents and eight publications reported 10 cases involving therapeutic agents. No discussion of the frequency of occurrence was presented. Osborne et al. reviewed nine published reports and found a mean frequency of 10.4% across 20 institutions (4). In their review of the literature, the NRC Subcommittee on Extravasation of the ACMUI found four studies that reported an average extravasation rate of 17% (1).

The ACMUI report suggests that a 17% extravasation rate is not consistent with reported rates for chemotherapy (0.09%) or IV contrast (0.24%) (1). Their report points out that the definition of extravasation is dependent on the agent—for radiopharmaceuticals it is “increased uptake of tracer at the injection site,” for non-radiopharmaceuticals it is “pain, swelling, or redness.” They report on one study that found that in 98% of the extravasation events, the amount of the activity at the injection site was less than 1% of the injected activity (5).

The literature on the sequalae of these events is very sparse. Reported injuries caused by extravasation of radiopharmaceuticals have been published occasionally as single case studies have [for examples, see (6–8)]. The ACMUI report “recognizes that, in rare cases, extravasated radiopharmaceuticals have caused serious tissue injuries to patients” (1).

This may account for the paucity of literature reports of injuries—although the extravasation event is easily detectable, the amount of activity in the tissue is too low to cause significant tissue damage. Another reason may be that serious tissue damage caused by ionizing radiation typically manifests itself many months or years after the event (9).

Even if an extravasation event only involves 1% of the injected activity, the radiation dose to the tissue could be substantial (hundreds to thousands of mGy), especially for therapeutic agents. Using data from Wilson et al. (10), (Table 1) below shows the estimated dose from a variety of diagnostic and therapeutic radiopharmaceuticals, assuming 1% of the injected activity.

**Table 1:** Estimated radiation dose to tissue if 1% of administered activity is extravasated.

Isotope	Label	Agent type	Self-dose factor (mGy/MBq)	Typical administered activity (MBq)^a^	Tissue dose (mGy)
C-11	Choline	Diagnostic	22.1	555^b^	122
F-18	Deoxyglucose	Diagnostic	270.3	278^c^	751
Ga-68	Dodatate	Diagnostic	126.9	140^d^	178
Tc-99m	Medronate	Diagnostic	18.8	555^e^	104
I-131	Iobenguane	Therapeutic	2,045.4	18,500^f^	378,399
Lu-177m	Dodatate	Therapeutic	3,958.6	7,400^g^	292,936

Adapted from Wilson et al. (10).

^a^ Based on package inserts (see below).

^b^ http://www.radiopharmaceuticals.info/uploads/7/6/8/7/76874929/c-11_choline_pi_2015_univ_tx.pdf.

^c^ https://marketing.webassets.siemens-healthineers.com/1800000006785434/7d48bf6cd7ae/1-14-2-2-Final-package-insert_1800000006785434.pdf.

^d^ http://www.radiopharmaceuticals.info/uploads/7/6/8/7/76874929/netspot_pi_-_jun_2021.pdf.

^e^ http://www.radiopharmaceuticals.info/uploads/7/6/8/7/76874929/mdp_cardinal_pi_2021.pdf.

^f^ https://www.azedra.com/content/pdf/full-prescribing-information.pdf.

^g^ https://www.accessdata.fda.gov/drugsatfda_docs/label/2018/208700s000lbl.pdf.

It should be noted here that the self-dose factors are based on a variety of assumptions. For example, data were generated for a water-filled 5 cm^3^ spherical volume of water containing a uniformly distributed activity of 100 MBq (10). However, the geometry of this model may be questionable since the skin and underlying muscles are composed of multiple layers. An alternative model might include a flattened disk, suggesting that the liquid may have infiltrated between layers. Also, although not stated, it appears that doses are calculated for the total lifetime of the isotope in question. No data is presented regarding the effects of the labelling compounds on migration outside the site or normal clearance from the tissue.

Assuming the radiopharmaceutical is administered intravenously into the antecubital vein, the main tissues at risk are the skin and underlying muscle tissue. It is known that radiation doses in excess of Gy (65,000 mGy) have a 50% risk of causing severe complications within five years (TD 5/50). For adult muscle tissue, the TD 5/50 is 80 Gy (80,000 mGy) (9). The data presented in (Table 1) suggests extravasations of diagnostic radiopharmaceuticals, even at 10 times the levels discussed, are unlikely to cause significant tissue damage. However, therapeutic agents are clearly a risk.

## Rationale for reporting extravasations

First, as discussed above, the data on the frequency of occurrence of extravasations is weak due to a lack of agreement on a definition. Mandatory reporting would increase the visibility of these events. It would also allow for collection of detailed data, including

• Radiopharmaceutical administered
• Prescribed route of administration
• Estimated activity remaining at the injection site or site of extravasation
• Root cause of extravasation
• Corrective and preventative actions taken to reduce the risk of reoccurrence
• Follow up to assess long-term consequences

Second, analysis of reported preventative actions taken would help develop best practices to prevent future problems.

Third, collection of data on long-term follow-up of the affected patients will help identify the level of activity that could be expected to cause significant tissue damage.

## Actions that can be taken now

### Acknowledgement

The first action is to acknowledge that extravasations can occur. The International Council on Radiation Protection (ICRP) Report 140 offers recommendations on preventing and detecting extravasations (11).

*Infusions must take place* *via* *an appropriate venous access device to ensure safe administration and prevent extravasation. Patients should be monitored for extravasation during administration. In the event of an extravasation, the infusion must be halted immediately … The event must be recorded, and follow-up is advised.*

The NRC ACMUI is on record opposing mandatory reporting of these events, due in large part to the estimated number that could be reported (1). They do, however, endorse the following efforts:

• Nuclear medicine facilities should have comprehensive quality control measures in place to monitor and track extravasations to improve the quality and safety of patients undergoing medical procedures involving the use of radiopharmaceuticals.
• While there should be a quality assurance policy to monitor and improve the extravasation rate at an institution, as there exists for many types of medical procedures, this should be conducted as part of a medical quality improvement initiative, and not subject to regulation by the NRC.

At a minimum, these events should be reported to a radiological or radiation therapy quality assurance committee and to senior management. An analysis of the root cause(s) should be initiated. Based on a review of the event, a plan of action should be initiated to prevent or reduce the risk of another occurrence.

The Radiation Safety Officer (RSO) is uniquely positioned within the institution to organize this effort. With access to all levels of management and personnel directly involved with administration of radiopharmaceuticals, the RSO is responsible for ensuring the safe use of radioactive materials, investigating events, and recommending and monitoring remedial actions (12). Senior management and clinical staff can be educated on the possibilities of an extravasation, how to detect it and its potential consequences. Wilson et al. offers practical tools to facilitate and standardize practical analysis of dosimetry for these events (10). The RSO and senior nuclear medicine clinical staff can develop appropriate policies for follow up on patients who may have a potentially serious extravasation.

### Training

At the operational level, staff training should include a discussion of identification of and risks of extravasations and what can be done to prevent them. For an illustration of the effectiveness of training, I offer the following example from my experience as an RSO. I have responded to and investigated multiple spills in nuclear cardiology departments. Almost all involved leakage from I.V. lines that were poorly connected, inadequately secured, or clogged. Contributing causes included administration by newly hired personnel (e.g., young physicians who were beginning their residency or fellowship in nuclear cardiology) and administration of the radiopharmaceutical through an existing I.V. line that had not been properly evaluated to ensure a secure connection and patency prior to beginning the injection. In collaboration with the chiefs of the nuclear cardiology departments and the chief nuclear medicine technologists, a training program was implemented that: (i) made staff aware of prior events and the root causes; (ii) emphasized starting new I.V. access; and (iii) flushing the line with saline prior to administration to ensure patency. This dramatically reduced the frequency of these events.

### Research

As discussed above, there is no agreement on the definition of a “significant” extravasation event. The ACMUI report states “There is no clinical evidence that patients are being harmed.” (1). This is short-sighted. Given that it is not standard clinical practice to follow patients, due in part to a lack of definition of these events, it is not surprising that there is little evidence, because there are few reports in the literature. More research is needed to better understand the nature of radiopharmaceutical extravasations. Questions include:

• How much activity is involved?
• Are there routes of administration that are more likely to result in extravasations?
• Are there scan types where extravasations are more likely?
• What is the appropriate model to be used to estimate tissue dose?
• How should the events be categorized in terms of seriousness of the injury? Should this scale be used to guide event reporting to the NRC?

The ACMUI reviewed five options for processes to trigger event reporting. The committee favored reporting those events requiring medical attention for a suspected radiation injury due to extravasation. This would not require dosimetry to be performed before reporting (1).

## Summary

Significant injuries from extravasation of radiopharmaceuticals are rare. However, there is no generally accepted definition of a significant extravasation, patients are not routinely followed, and radiation injuries can take months to years to appear. These complications demonstrate the need for further action and research. Steps can be taken by radiation protection personnel at medical institutions to educate practitioners and staff about extravasations, their risks and what steps can be taken to reduce their occurrence. Further research needs to be performed to better understand the incidence rate, categorization, causes, and methods of prevention.

## Data Availability

The original contributions presented in the study are included in the article/Supplementary Material, further inquiries can be directed to the corresponding author/s.
